# Electrostatic Tuning of Bilayer Graphene Edge Modes

**DOI:** 10.3390/nano13142102

**Published:** 2023-07-19

**Authors:** Hira Ali, Llorenç Serra

**Affiliations:** 1 Institute for Cross-Disciplinary Physics and Complex Systems IFISC (CSIC-UIB), E-07122 Palma, Spain; 2 Physics Department, University of the Balearic Islands, E-07122 Palma, Spain

**Keywords:** bilayer graphene, edge modes, electric transport

## Abstract

We study the effect of a local potential shift induced by a side electrode on the edge modes at the boundary between gapped and ungapped bilayer graphene. A potential shift close to the gapped-ungapped boundary causes the emergence of unprotected edge modes, propagating in both directions along the boundary. These counterpropagating edge modes allow edge backscattering, as opposed to the case of valley-momentum-locked edge modes. We then calculate the conductance of a bilayer graphene wire in presence of finger-gate electrodes, finding strong asymmetries with energy inversion and deviations from conductance quantization that can be understood with the gate-induced unprotected edge modes.

## 1. Introduction

**Figure 1:**
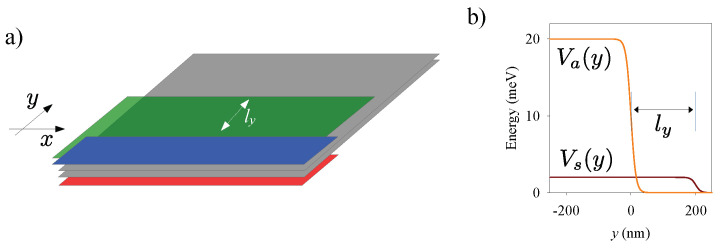
(**a**) Sketch showing the two bilayer graphene planes (gray), the pair of electrodes for asymmetric potential $V_{a}$ (red and blue) and the electrode for symmetric potential $V_{s}$ (green). A *y*-displacement of the symmetric and asymmetric electrodes is indicated by $l_{y}$. (**b**) Model symmetric $V_{s}\left(y\right)$ and asymmetric $V_{a}\left(y\right)$ potentials with a displacement $l_{y}=200$ nm, $V_{s}^{0}=2$ meV, $V_{a}^{0}=20$ meV.

Bilayer graphene (BLG) allows a remarkable mechanism of electronic confinement by tuning the energy gap with electrostatic gates on the sides of the two graphene layers [1,2,3,4,5,6,7,8,9,10]. Indeed, an interlayer electric field opens a gap in the spectrum, thus favouring electronic confinement to those regions with a vanishing (or small) interlayer field. Electrodes of carefully chosen shapes, designed with lithographic techniques, allow different types of BLG nanostructures such as open semi-infinite edges, quasi-1D wires (electron guides), and fully closed loops, rings and dots. For instance, the blue/red electrodes in Figure 1a create an open BLG edge at y=0, separating two half planes, gapped and ungapped, for electronic motion.

Graphene nanostructures can also be made with etching techniques, removing parts of the graphene system, as opposed to the above mentioned electrostatic confinement of BLG. With etching, however, the specific atomic arrangement at the borders as well as the presence of undesired edge roughnesses or imperfections is usually relevant and methods to reduce or minimize them are generally desired [11,12,13].

The tuning of the electric gap in BLG using electric fields was demonstrated in early magnetotransport experiments with bulk BLG [14]. These were followed by a very intense research activity, as summarized, e.g., in the field reviews Refs. [1,3]. More recently, experiments on electrostatic confinement in BLG nanostructures have been reported for dots [6,7,8,9,10] and 1D edges [12,15,16,17,18]. The hallmark of the latter are the observation of conductance quantization in quantum transport experiments.

Ungapped BLG hosts bulk propagating electronic modes for any energy, with characteristic 2D wave numbers, (k,q) in (x,y) directions. States in translationally invariant edges or wires in only one direction (*x*) have a 1D wave number (*k*); while closed loops and dots possess a fully discrete electronic spectrum. In Ref. [19] it was shown that an open electrostatic edge in BLG is able to bind an edge mode with a characteristic valley-momentum locking; i.e., with opposite valleys propagating in opposite directions along the edge. Remarkably, the wave number *k* of this mode separates from the continuum band of bulk ungapped modes and yields characteristic transport signatures in BLG junctions [19].

In this work we further investigate the properties of electrostatic edge modes in BLG. In particular, we focus on the effect of a potential shift as induced by an additional side electrode. We consider an electrostatic edge defined by two side gates (red and blue in Figure 1a), and an additional gate creating the potential shift (green in Figure 1a). We found that a lateral shift of the electrodes by a small distance $l_{y}$ has a very relevant effect. It causes additional edge modes in the stripe of width $l_{y}$, running in both directions along the edge. Therefore, they allow backscattering mediated by these edge modes alone, without the need to couple with bulk modes. In presence of disorder, or other inhomogeneities, an additional electrode will then strongly affect the conductance along the edge. Besides, the electrode also causes energy-inversion asymmetry, with different conductances for positive and negative energies (with zero energy being the Dirac-point reference energy).

Subsequently, we use the results on the open edge to understand the effect of finger gate electrodes (FGE) across a BLG wire or guide. We consider the cases of an extended FGE covering both wire edges, or shorter FGE’s affecting one or the two edges of the wire. We predict conspicuous energy asymmetries and conductance deviations from quantization that can be explained with the FGE induced edge modes. Therefore, similarly to the case of semiconductor wires [20], FGE’s are a practical way to manipulate electronic transport in BLG electrostatic wires.

## 2. Theoretical Model

Our analysis is based on a low-energy Hamiltonian for BLG in presence of electrostatic potentials [1]. We consider two types of potentials: a symmetric potential $V_{s}$, equal on the two layers, and an asymmetric potential $\pm V_{a}$, with opposite signs on the two layers. In this work we consider parameterized model functions for both potentials, as shown in Figure 1b for the case of an open edge. These functions read

(1) $$V_{s/a}\left(y\right)=\frac{V_{s/a}^{0}}{1+e^{(y-y_{s/a})/s}}\;,$$

where parameters $V_{s/a}^{0}$ and $y_{s/a}$ are the asymptotic value and position of the border for the symmetric/asymmetric potential. Parameter *s* is a small distance representing the smoothness of the potential steps. Examples of our model potentials can be seen in Figure 1b.

The low-energy effective Hamiltonian we will use in this work is built on an underlying tight-binding atomistic description for BLG. The electronic band struture of unbiased bulk BLG is characterized by gap closings at the six Dirac points in reciprocal space, three of them corresponding to the valley $K_{+}$ and the other three to valley $K_{-}$. Near those Dirac points, an expansion to the leading terms in electronic momenta yields an effective multiband continuum Hamiltonian. We refer the reader to Ref. [1] for details on the mathematical derivations and only stress here that we restrict to graphene layers in AB Bernal stacking. Adding the model potentials of the type (1) to the resulting effective Hamiltonian describes the specific confinement mechanisms due to the electrostatic gates of this work. The potential difference $2V_{a}$ between the two graphene layers opens an energy gap in the low-energy scale around the Dirac points which is modulated in space by a position dependent potential.

The BLG low-energy Hamiltonian reads [1]

(2) $$\begin{array}{ccc} H & = & v_{F}p_{x}\tau_{z}\sigma_{x}+v_{F}\;p_{y}\sigma_{y}+\frac{t}{2}\;\left(\;\lambda_{x}\sigma_{x}+\lambda_{y}\sigma_{y}\;\right)\\ & + & V_{s}\left(x,y\right)+V_{a}\left(x,y\right)\;\lambda_{z}\;, \end{array}$$

with the Fermi velocity $\hbar v_{F}=660\;\mathrm{meV}\;\mathrm{nm}$ and the interlayer coupling t=380meV. In Equation (2), $\sigma_{x,y,z}$, $\tau_{x,y,z}$ and $\lambda_{x,y,z}$ are sets of Pauli matrices for sublattice, valley and layer degrees of freedom, respectively. This Hamiltonian is valley diagonal and it has been used to study quantum states in a variety of BLG nanostructures. As mentioned in Section 1, the use of position-dependent potentials $V_{s}\left(x,y\right)$ and $V_{a}\left(x,y\right)$ allow modeling the effect of potential gates that create electrostatic borders. Notice that Equation (2) is for general inhomogenous potentials $V_{s}$ and $V_{a}$ depending on both coordinates (x,y). See below, however, for the restricted cases considered in this work of potentials which are uniform along *x* or piecewise-uniform along *x*, with each uniform section of the type given in Equation (1).

We also stress that for the case of sign-changing $V_{a}\left(x,y\right)$’s, Hamiltonian (2) predicts the emergence of topological modes near the sign-change border. The spectrum becomes gapless in presence of these modes since their energies E(k) cross from the negative to the positive energy sectors. These modes also show a characteristic valley-momentum locking and protection from bulk modes by an energy gap [21,22,23,24,25,26]. From a formal Condensed Matter topology approach, it has been pointed out that specific invariants for each valley $N_{\tau }=\pm 1$ can be approximately defined in BLG [2,27]. However, it has also been stressed that the bulk-boundary correspondence between those invariants and the edge modes is not general and may depend on the specific type of interface, such as in BLG-vacuum or BLG-BLG [27]. The latter type corresponding to the electrostatic boundaries considered in this work.

Below, we will discuss (a) the eigenstates of the Hamiltonian (2) for fully translational invariant BLG systems with both potentials $V_{s}\left(y\right)$ and $V_{a}\left(y\right)$; and (b) the conductance through junctions of different BLG sections described by $V_{s}\left(x,y\right)$ and $V_{a}\left(x,y\right)$ having a piecewise-constant dependence on *x*. They model the effect of a central FGE on a quantum wire (see device sketches in Figures 3–5). In all cases our resolution method is based on a combination of spatial grid discretization and multiple component wave functions using complex-band-structure theory. More details of the method can be found in Section 5.

## 3. Results and Discussion

### 3.1. Single Edge

**Figure 2:**
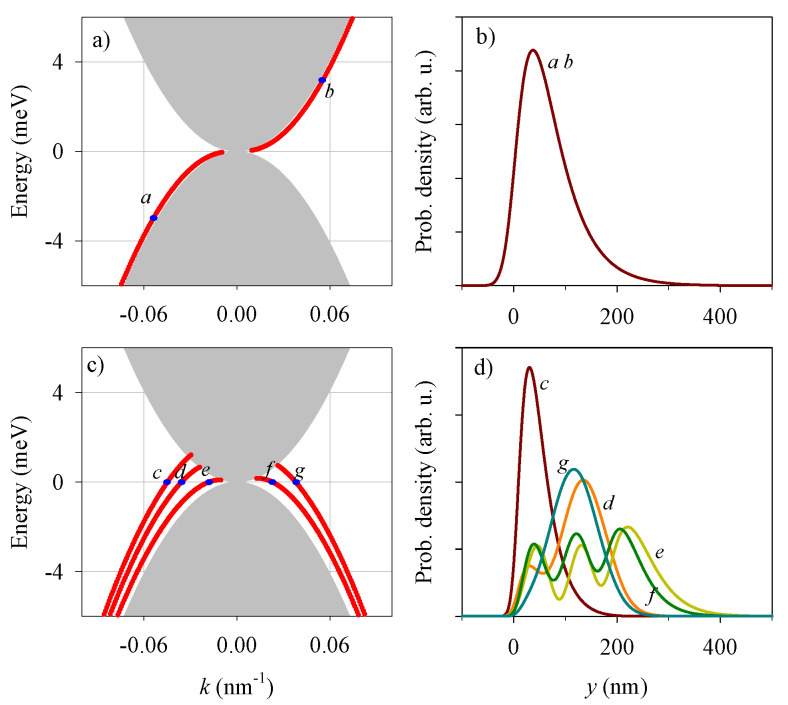
(**a**) Eigenenergies for the (open) single edge with $V_{s}=0$. The gray region is the bulk continuum while the red line is the discrete branch of edge states. (**b**) Spatial probability distributions for two selected wave numbers *k* indicated in panel (**a**) by the corresponding labels. (**c**,**d**) Similar results to (**a**,**b**) but with $V_{s}=2$ meV and $l_{y}=200$ nm. Other parameters: $V_{a}=20$ meV, s=7.5 nm.

Figure 2 shows the electron eigenenergies for the open BLG edge sketched in Figure 1. The gray region in Figure 2a is the continuum for bulk modes, given by the condition

(3) $$|k|<\frac{1}{\hbar v_{F}}\;\sqrt{|E|\;\left(\;|E|+t\;\right)}\;.$$

See Appendix A for a derivation of this momentum restriction for bulk propagating states. The red line in Figure 2a shows the edge mode in absence of symmetric potential $V_{s}=0$. This mode spatially decays with the distance to the boundary (Figure 2b) and it is characterized by valley-momentum locking; reversed valleys propagating in reversed directions in a similar way to the quantum spin Hall effect but replacing spin with valley.

The edge mode becomes damped when it overlaps with the continuum of bulk BLG modes, indicated in gray colour in Figure 2a. In this case the localized edge mode decays into bulk modes with the same *E* and *k*, thus flying away from the edge. In Figure 2a this overlap occurs in the region of vanishing *E* and *k* and, technically, it is not easily resolved by the numerical calculation.

The modification induced by a potential shift of an additional gate with $l_{y}=200$ nm is shown in Figure 2c,d. The discrete branch of states of Figure 2a is now shifted upwards in energy, merging with the continuum for energies beyond a given maximum value. In addition, new branches of modes emerge at low and negative energies that are localized to the region of width $l_{y}$ near the boundary. These modes propagate in both directions, as seen from the positive and negative slopes of the energy branches. The corresponding probability densities show substantial overlaps (Figure 2d), suggesting the possibility of backscattering mediated by these edge modes in presence of inhomogeneities along the edge. Most remarkably, the additional side gate (and potential shift $V_{s}$) yields energy-inversion asymmetry in Figure 2c, with the presence of edge-mode branches only in the lower part of the energy diagram. We stress that the shift $l_{y}$ in Figure 1a is essential for the emergence of additional branches of edge states, as well as for the energy asymmetry of the spectra.

### 3.2. Quantum Wire Junctions

**Figure 3:**
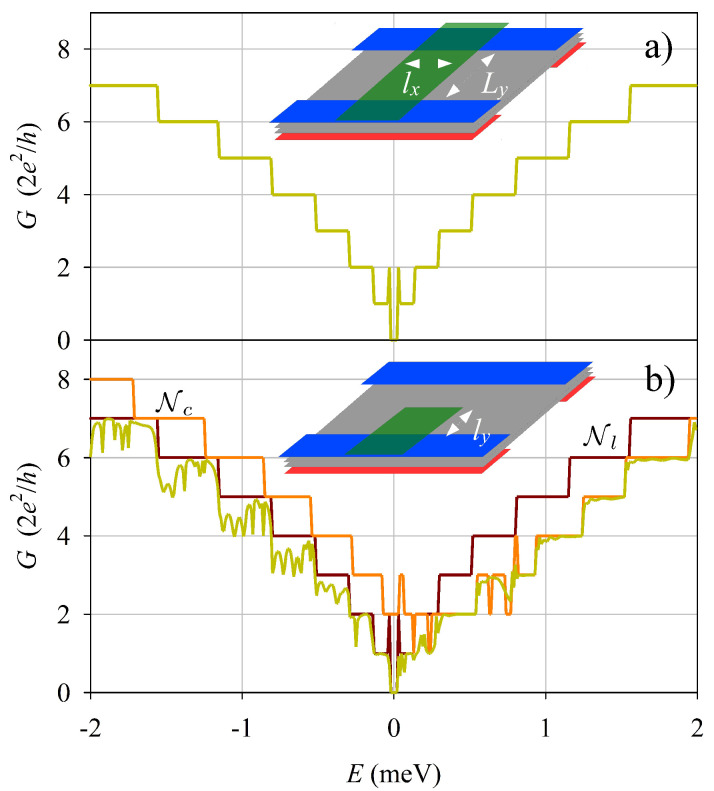
(**a**) Conductance of a quantum wire in presence of a FGE across all the wire (**a**), and with a FGE covering only one edge (**b**). The sketch insets show the corresponding systems. The number of active modes in the asymptotic leads $N_{l}$ and center $N_{c}$ are also shown. Parameters: $L_{y}=600$ nm, $l_{y}=200$ nm, $l_{x}=1$ μm, $V_{s}^{0}=0.5$ meV.

**Figure 4:**
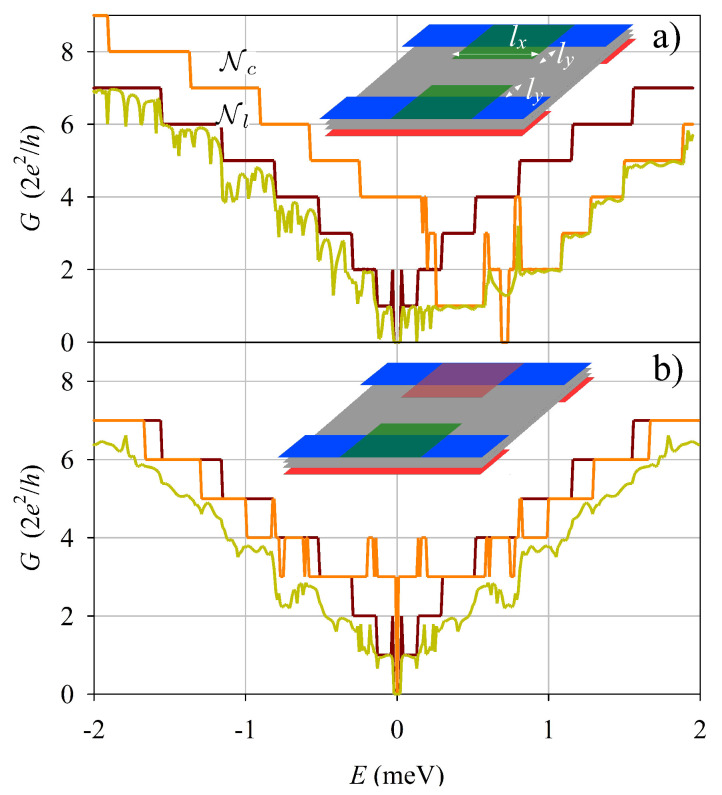
Similar to Figure 3 but with two FGE’s with the same $V_{s}^{0}$ (**a**), and with opposite $V_{s}^{0}$ (**b**).

**Figure 5:**
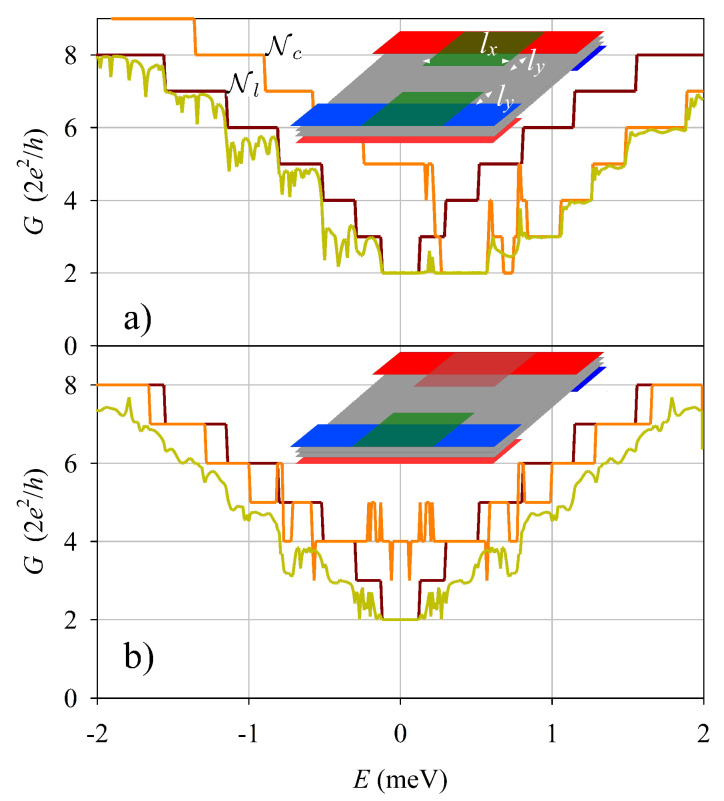
Similar to Figure 3 and Figure 4 but for a quantum wire with topological confinement; i.e., with reversed blue and red electrodes on the two wire edges, as shown in the inset sketches.

Having analyzed the gate-induced modifications in the open edge, we consider next the role of a FGE on an electrostatic quantum wire of width $L_{y}$. More specifically, we calculate the total left-to-right transmission *T* (with conductance $G=Te^{2}/h$) using the complex-band-structure method for the double junction system sketched in Figure 3, Figure 4 and Figure 5. Firstly, Figure 3a shows that a FGE covering all the wire has a negligible effect on the wire conductance: transmission is perfect and the coductance simply reproduces the staircase function of the number of active modes. On the contrary, a FGE covering only one edge of the quantum wire (Figure 3b) yields relevant modifications. *G* deviates from the plateau values, with conspicuous minima for energies $E<V_{s}^{0}$. There is also a clear asymmetry with respect to energy inversion in Figure 3b. For $E>V_{s}^{0}$ the conductance is almost perfectly quantized, while for $E<V_{s}^{0}$ it shows the mentioned deviations.

The conductance non-quantization and asymmetry of Figure 3b can be understood as effects of the edge modes induced by the FGE, as discussed above. Quasi bound states, allowed by edge mode backscatterings at the interfaces, lead to conductance dips for specific (resonant) energies. This mechanism is only present for $E<V_{s}^{0}$, thereby explaining the asymmetry in conductance.

The case of two FGE’s, one on each edge of the wire, is presented in Figure 4. We studied this configuration using the same shift $V_{s}^{0}$ on the two FGE’s (Figure 4a), and with opposite signs of the shift $\pm V_{s}^{0}$ on the two FGE’s (Figure 4b). The case of identical shifts is very similar to the preceding case with just a single FGE (Figure 3b). However, with opposite signs the results change markedly; the conductance becoming again symmetric with energy inversion and the deviations from quantization are enhanced.

As a final case, we consider a topological inversion in the asymmetric potential $V_{a}$ [28,29,30,31,32], with the red/blue electrodes being reverted on the two edges of the quantum wire (Figure 5) and with two FGE’s. The results in Figure 5a,b are very similar to those in Figure 4a,b but with a notable difference near zero energy. Namely, in the topological cases the conductance is perfectly quantized to $4e^{2}/h$ in a small energy plateau around zero, while it vanishes in Figure 3 and Figure 4. This is explained by the gapless character of the topological wire, which hosts two valley-momentum-locked branches crossing zero energy. On the contrary, the nontopological (trivial) confinement in a finite $L_{y}$ wire is always characterized by a zero energy gap due to the finite size.

The energy inversion symmetries of the different configurations of FGE electrodes considered in Figure 4 and Figure 5 is summarized in Table 1. Notice that this symmetry is fixed by the product of the signs of FGE potentials on opposite edges of the wire, irrespectively of the trivial or topological character of the wire confinement. This result illustrates how conductance measurements could be used to observe tuning of the edge modes using FGE’s.

### 3.3. Further Discussion

All results presented above are for a single valley, $K_{+}$. The corresponding results for the reversed valley $K_{-}$ can be inferred simply reverting k→−k in Figure 2a,b for a single edge, while the results remain invariant in the cases of wires with FGE’s of Figure 3, Figure 4 and Figure 5. Therefore, we do not find any valley polarization induced by a FGE in the quantum wire.

An important underlying aspect, however beyond our present analysis, is the role of random imperfections and disorder in the device. While it can be reasonable to assume that BLG is relatively free of such disorder effects, the additional processing required for the electrostatic electrodes could introduce such random disorder. Therefore, this is a relevant aspect to consider in the future. We may expect, however, that the conductance asymmetry and the nonquantization induced by FGE’s in a quantum wire would be enhanced in presence of random disorder.

## 4. Conclusions

We have studied the role of an electrode creating a potential shift near an electrostatic BLG edge. We found that in presence of a displacement $l_{y}$ between the BLG edge and the additional electrode, new modes emerge near the edge, in the region of width $l_{y}$, that propagate in both directions. Furthermore, the valley-momentum-locked branch of the single edge is shifted in energy by the electrode and the spectrum becomes asymmetric with energy inversion.

We also investigated the more practical case of a BLG quantum wire in presence of transverse FGE’s. If a FGE is covering the two edges of a quantum wire, the system’s conductance is almost unchanged and remains nearly perfectly quantized. However, if the FGE covers only one edge, or there are two different FGE’s covering the two edges, then the conductance displays strong non quantizations and asymmetries with respect to energy inversion. These changes are in good agreement with the modifications expected from the single-edge spectrum in presence and a displaced electrode. The energy inversion symmetry in the quantum wire is restored with two FGE’s having opposite potential signs on the two edges. In summary, our work shows that FGE’s can be a practical way to manipulate transport properties of BLG quantum wires by the electrostatic tuning the electronic modes at the wire edges.

## 5. Methods

We solved the eigen-problem with Hamiltonian (2) using finite-difference discretization and matrix diagonalization routines. With translational invariance, in the cases of a single edge and quantum wire, a matrix diagonalization for each (real) wave number *k* yields the band structure $E_{n}\left(k\right)$ as well as it corresponding eigenstates. An important aspect is the filtering of spurious modes emerging due to an artificial Fermion doubling of the physical eigenstates. In practice the filtering is done by eliminating those states with large oscillations in neigbouring grid points, such that spatially averaging on a small neigbourhood strongly modifies the wave function. We found this simple technique to be quite effective and robust [31].

The transport problem for the junctions of piece wise homogenous sections in the transport direction (*x*) was solved using the complex-band-structure approach discussed in Ref. [33]. Here, it is important to include complex wave numbers *k* in order to describe evanescent-state behavior in the proximity of the junction interfaces. The wave-function matching at the junction interfaces is transformed into a large set of linear equations whose solution determines the quantum transmissions $T_{kk^{\prime }}$ and its corresponding Landauer conductance $G=\frac{e^{2}}{h}\sum_{kk^{\prime }}T_{kk^{\prime }}$. We refer to Ref. [33] for more details of the complex band structure approach and to Refs. [19,31] for its specific application to BLG structures.

## Figures and Tables

**Table 1:** Symmetric/antisymmetric (S/A) character of the conductances in Figure 4 and Figure 5. The column FGE1 × FGE2 indicates the sign product of the FGE potentials covering the two wire edges.

Confinement	FGE1 × FGE2	Conductance
Trivial	+	A
	−	S
Topological	+	A
	−	S

## Data Availability

Computer codes used to obtain the numerical results are available upon request to the authors.

## Appendix A. Bulk Continuum

For constant $V_{s/a}$ potentials the eigenstates of the Hamiltonian (2) are plane waves, with momenta (k,q) along (x,y),

(A1) $$\Psi \equiv \Phi_{\eta_{\sigma }\eta_{\tau }\eta_{\lambda }}\;e^{i(kx+qy)}\;,$$

with $\eta_{\sigma },\eta_{\tau },\eta_{\lambda }=1,2$ indicating the different spinorial components of the wave function. Assuming a given real *k*, we can determine the corresponding *q*’s by transforming the eigenvalue equation as follows

(A2) $$H\Psi =E\Psi \;\Rightarrow \;\sigma_{y}H\Psi =E\sigma_{y}\Psi \;.$$

The purpose of the above transformation is that Equation (A2) can be easily rewritten as an eigenvalue equation for *q*,

(A3) $$\begin{array}{ccc} \frac{1}{\hbar v_{F}}\left[E\;\sigma_{y}+i\;\hbar v_{F}\;k\;\sigma_{z}\tau_{z}\right. & + & \frac{t}{2}\left(i\;\lambda_{x}\sigma_{z}-\lambda_{y}\right)\\ & - & V_{s}\;\sigma_{y}-\left.V_{a}\;\sigma_{y}\lambda_{z}\right]\Phi =q\;\Phi \;. \end{array}$$

After some algebra the eigenvalues of Equation (A3), assuming free BLG for which $V_{s/a}=0$, can be analytically determined with the diagonalization of an algebraic matrix. The *q* eigenvalues read

(A4) $$q=\pm \frac{1}{\hbar v_{F}}\sqrt{-\hbar^{2}v_{F}^{2}\;k^{2}+\left|E\right|\left(\;|E|\pm t\;\right)}\;.$$

Notice that Equation (A4) already proves the existence of a critical value $k_{c}=\sqrt{\left|E\right|\left(\;|E|+t\;\right)}/\hbar v_{F}$, as given in Equation (3). Indeed, the propagating-mode condition requires *q* to be real which, from the square root in Equation (A4), requires $|k|<k_{c}$.
